# Increased Incidence of Thrombotic Complications With Non-small Cell Lung Cancer Necessitates Consideration of Prophylactic Anticoagulation in Young Individuals

**DOI:** 10.7759/cureus.17769

**Published:** 2021-09-06

**Authors:** Arindam Bagchi, Mohammad Saud Khan, Arti Saraswat, Affan Ansari, Qiang Nai, Veena Iyer, Danae Hamouda, Sadik Khuder, Cherian Verghese

**Affiliations:** 1 Oncology, Beckley Appalachian Regional Healthcare (ARH) Hospital, Beckley, USA; 2 Cardiology, University of Kentucky College of Medicine, Bowling Green, USA; 3 Internal Medicine, University of Kentucky College of Medicine, Bowling Green, USA; 4 Internal Medicine, Vasantrao Pawar Medical College and Hospital, Nashik, IND; 5 Oncology, Massachusetts General Cancer Center, Boston, USA; 6 Oncology, Brigham and Women Hospital, Boston, USA; 7 Oncology, University of Toledo Medical Center, Toledo, USA; 8 Internal Medicine, University of Toledo Medical Center, Toledo, USA

**Keywords:** oncology, venous thromboembolism (vte), pulmonary embolism (pe), deep venous thrombosis (dvt), mortality, metastatic non-small cell lung cancer

## Abstract

Venous thromboembolism (VTE) is a common complication in cancer patients and is associated with increased morbidity and mortality. Lung cancer is commonly associated with VTE including pulmonary embolism. We did a retrospective analysis from the 2013 Healthcare Cost and Utilization Project data to determine the role of age as a factor in the development of VTE in this patient group. Patients were selected using the International Classification of Diseases, Ninth Revision (ICD-9) diagnosis codes for metastatic lung cancer and VTE. The patients were stratified by age, sex, race/ethnicity, and site of VTE. There was a total of 16,577 VTE events detected out of a total of 182,863 cases of metastatic lung cancer, representing 9% of the total cases. In patients under 65 years of age, there were 356.82 more cases of pulmonary embolism per 100,000 individuals compared to those older than 65 years (p<0.0001). The same age group also showed 374.83 more upper extremity VTE, 286.94 more non-pulmonary thoracic VTE, and 263.97 more abdominal VTE events per 100,000 individuals (p<0.0001). In conclusion, we found that patients under the age of 65 years had a significantly higher incidence of VTE, pulmonary embolism, upper extremity VTE as well as abdominal and non-pulmonary VTE.

## Introduction

Venous Thromboembolism (VTE) is a common complication in cancer patients. The incidence of VTE in the general population is about 1-3% per 1000 per year [1]. However, this risk increases significantly and is four to seven times higher in cancer patients [2,3]. And this is associated with poor survival due to its significant effect on mortality and morbidity. More specifically the diagnosis of VTE has been associated with a higher risk of death within two years for non-small cell lung cancer (NSCLC) and small cell lung cancer (SCLC) [1].

The incidence of VTE in lung cancer varies from 3-13.9% depending on the study [4,5]. Adenocarcinomas have a three-fold higher risk as compared to squamous cell carcinoma for development of VTE even after adjustment for age, sex, cancer treatment, and stage [6]. Also, patients with advanced cancer stage and distant metastases are more likely to develop complications in the form of VTE. Chemotherapy and radiation therapy both have been associated with an increased risk of VTE [7]. It is likely linked to direct endothelial damage and down-regulation of the coagulation pathway. Analyses have found that the risk for VTE increased three-folds on starting chemotherapy in presence of the already increased risk due to lung cancer, with an increase in risk with the duration of chemotherapy [8]. Among the lung cancer patients receiving chemotherapy, the majority of VTE occurs within six months of starting chemotherapy [7-9].

Age as a risk factor for VTE in lung cancer patients

Age per se, as a risk factor for VTE in lung cancer patients, has not been studied very extensively. Some prior studies evaluating age as a risk factor for VTE in all cancer subtypes found that cancer-associated VTE occurred more frequently in older age groups [10].

Based on this background, we looked at the incidence of VTE in metastatic lung cancer patients and determine the role of age as a factor in the development of VTE in this patient group. We did a retrospective analysis from the 2013 Healthcare Cost and Utilization Project (HCUP) data.

## Materials and methods

The National Inpatient Sample (NIS) is a set of longitudinal hospital inpatient databases included in the HCUP database. These databases are created by Agency for Health Research and Quality (AHRQ) through a Federal-State-Industry partnership. It includes data from more than seven million hospital stays each year and when weighted, it estimates more than 35 million hospitalizations nationally.

We used the HCUP dataset for the purpose of our analysis. Being a publicly available dataset, prior protocol review and approval from the local or regional biomedical and ethical Institutional Review Board (IRB) was not mandated for the purpose of our study and data analysis.

Patients were selected using ICD-9 diagnosis codes for metastatic lung cancer (including all possible metastatic sites that could have been captured by the diagnosis codes, including metastases to liver, lung, bones, brain, adrenals, skin, and soft tissue) and VTE. Diagnoses of VTE were then stratified by site, including upper extremity, lower extremity, pulmonary, abdominal, and non-pulmonary thoracic VTE. The patients were also stratified by age, sex, race/ethnicity. The differences in the incidence of VTE among the groups were calculated and analyzed using the Statistical Analysis System (SAS®) software version 9.4 (SAS Institute Inc 2013, Cary, NC) and Chi-square tests.

## Results

There was a total of 16,577 VTE events detected out of a total number of 182,863 cases of metastatic lung cancer, representing 9% of the total cases. Further, subgroup analyses showed that in patients under 65 years of age, there were 356.82 more cases of pulmonary embolism per 100,000 individuals compared to those older than 65 years (p<0.0001). The same age group also showed 374.83 more upper extremity VTE, 286.94 more non-pulmonary thoracic VTE, and 263.97 more abdominal VTE events per 100,000 individuals (p<0.0001). There was no statistically significant difference in the incidence of lower extremity VTEs between the sub-groups (Table 1).

**Table 1:** Incidence of VTE in lung cancer patients stratified based on their age (<65 years and ≥ 65 years). VTE: Venous thromboembolism

Characteristic	Age less than 65 years	Age more than or equal to 65 years	p-value (by Chi-square test)
Total number of patients (n)	77,415	105,448	
Pulmonary embolism	2928 (3782.21 events per 100,000 persons)	3612 (3425.38 events per 100,000 persons)	< 0.0001
Upper Extremity VTE	1064 (1178.06 events per 100,000 persons)	998 (803.23 events per 100,000 persons)	<0.0001
Lower Extremity VTE	2791 (3605.24 events per 100,000 persons)	3913 (3710.83 events per 100,000 persons)	0.2352
Non-Pulmonary Thoracic VTE	460 (594.20 events per 100,000 persons)	324 (307.26 events per 100,000 persons)	<0.0001
Abdominal VTE	324 (418.52 events per 100,000 persons)	163 (154.57 events per 100,000 persons)	< 0.0001

Stratifying by ethnicity, African-Americans had 848.0 more lower extremity VTE events (p < 0.0001), 599.23 more pulmonary embolism events (p < 0.0001), 468.81 more upper extremity VTE events (p < 0.0001), 259.94 more non-pulmonary thoracic events (p < 0.0001) and 148.41 more abdominal VTE events (p < 0.0001), as compared to Caucasians (Table 2).

**Table 2:** Incidence of VTE in lung cancer patients stratified based on their ethnicity (African-Americans and Caucasians). VTE: Venous thromboembolism

Characteristics	African American	Caucasian	p-value (by Chi-square test)
Pulmonary embolism	996 (4121.4 events per 100,000 persons)	4508 (3522.2 events per 100,000 persons)	< 0.0001
Upper extremity VTE	331 (1369.6 events per 100,000 persons)	1153 (900.8 events per 100,000 persons)	< 0.0001
Lower extremity DVT	1062 (4396 events per 100,000 persons)	4541 (3548 events per 100,000 persons)	< 0.0001
Non-pulmonary Thoracic VTE	156 (645.5 events per 100,000 persons)	494 (385.5 events per 100,000 persons)	< 0.0001
Abdominal VTE	91 (376.5 events per 100,000 persons)	292 (228.1 events per 100,000 persons)	< 0.0001

## Discussion

We analyzed the incidence of VTE in lung cancer patients stratified based on their age with one group being less than 65 years and the other group being equal to or more than 65 years of age. We found that patients under the age of 65 years had significantly higher incidence of VTE, pulmonary embolism, upper extremity VTE as well as abdominal and non-pulmonary VTE.

We also found that when stratified by race/ethnicity, African-Americans were found to have a higher incidence of VTEs as compared to Caucasians. This is consistent with other data sets and reports, indicating racial/ethnic disparities in incidence and mortality among advanced-stage lung cancer patients [11,12]. One possible explanation of this increased incidence of VTE in younger patients can be the biological aggressiveness of lung cancer, leading to an increased thrombotic risk in this age group. Associations have been observed with one-year relative mortality as a measure of biological aggressiveness of the cancer and an associated thrombogenic potential [13].

Patients with adenocarcinoma have the highest risk for developing VTE as compared to other histological subtypes. Blom et al. studied a cohort of 537 patients with a first diagnosis of lung cancer; adenocarcinomas had a three-fold higher risk as compared to squamous cell carcinomas for development of VTE even after adjustment for age, sex, cancer treatment, and stage [2]. Other datasets have also identified VTEs occurring more frequently in adenocarcinoma as compared to squamous cell carcinoma [14]. The increased incidence of VTE in adenocarcinomas is hypothesized to be related to mucin production and subsequent activation of platelets and pro-coagulant factors.

Adverse factors increasing the risk for VTEs also include advanced stage of tumor, administration of chemotherapy, type of chemotherapy involved, and presence of other comorbidities. Histopathological differences also contribute to associated VTE frequency.

The clinical benefit of knowing which age group would be at an increased risk of developing VTE may further help in risk stratifying patients for prophylactic anticoagulation. Past studies also have postulated prediction models for chemotherapy-associated VTE, which do take into account the cancer type, being very high (stomach or pancreas cancer) or high (lung, gynecologic, testicular, bladder cancers, or lymphoma) risk, along with other factors such as body mass index, prechemotherapy cell counts, etc [15]. However, age per se has not been reported to be a part of these models.

In addition to the factors postulated in the predictive models, taking into consideration other high-risk factors such as adenocarcinoma histology, stage, and first six months of chemotherapy administration may help in identifying patients who may benefit from prophylactic anticoagulation and, thus, reducing VTE-associated morbidity and mortality.

The study has several limitations stemming from the utilization of an administrative inpatient database. This database is based on diagnostic and procedural codes thus there can be variation in coding practices among different hospitals and personnel. The NIS database does not allow us to determine the treatment regimen that the patients were on for the malignancy and for the VTE. NIS data also cannot differentiate between the readmission entries.

## Conclusions

In conclusion, we found that lung cancer patients under the age of 65 years had a significantly higher incidence of VTE, pulmonary embolism, upper extremity VTE as well as abdominal and non-pulmonary VTE and, therefore, may benefit from prophylactic anticoagulation. Also, there was a higher incidence of VTE including pulmonary embolism, non-pulmonary thoracic VTE, abdominal VTE, upper and lower extremity VTE in the African-American population as compared to Caucasians. 
